# The forearm fillet flap: ‘spare parts’ reconstruction for forequarter amputations^*^

**DOI:** 10.1080/23320885.2019.1666718

**Published:** 2019-09-20

**Authors:** Haripriya S. Ayyala, Omar M. Mohamed, Paul J. Therattil, Edward S. Lee, Jonathan D. Keith

**Affiliations:** Division of Plastic Surgery, Department of Surgery, Rutgers New Jersey Medical School, Newark, NJ, USA

**Keywords:** Microsurgery, free flap, fillet flap, limb salvage, sarcoma

## Abstract

Radical forequarter amputation is often performed for recurrent proximal extremity tumors. A free forearm fillet flap is used to provide excellent coverage of the resultant defect without donor site morbidity. Use of a free flap from the distal portion of the extremity with proximal tumor burden is safe and effective.

## Introduction

The creation of a donor site from a free flap is not without morbidity, especially in a patient already undergoing major extirpative surgery. The free fillet flap may be a practical option when reconstructing this type of defect in an extremity. This flap is often characterized using the ‘spare parts’ notion, which employs the principle of utilizing viable and undamaged tissue from amputated limbs that would otherwise be discarded. These flaps are most commonly utilized after severe traumatic injuries to the extremities where replantation is contraindicated, however, their description in the setting of malignant neoplasms is lacking [1–8].

Although fillet flaps are more commonly implemented in reconstruction of the lower extremity [4,6,7], their successful use in the hand, forearm and shoulder have been reported [1,2,4,5,9]. This is likely because of narrower replantation indications for the lower extremity [3,4] or the high frequency of limb sparing tumor resections in the upper extremity [10]. Nevertheless, there are several benefits to fillet flaps including elimination of donor site morbidity and immediate coverage of the wound [2,6,11]. The authors describe the use of this flap in salvage forequarter amputations for recurrent osteosarcoma of the upper extremity.

## Case I

**Figure 1.**
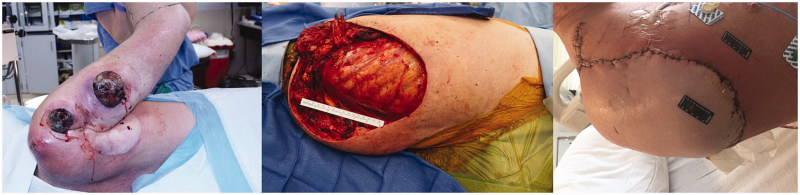
Case I pre-operative mass, intra-operative defect and post-operative healed flap .

A 57-year-old man presented for salvage resection of a recurrent osteosarcoma of the right shoulder and chest wall (Figure 1). He had previously undergone neoadjuvant radiation therapy followed by resection and coverage with a free anterolateral thigh flap anastomosed to the thoracodorsal vessels. On physical exam, the patient had two large fungating masses overlying the right proximal shoulder. Magnetic resonance imaging revealed the masses to be in close proximity to the axillary vessels with metastases to the lungs. In order to decrease tumor burden and increase the patient’s quality of life, a forequarter amputation was planned. Intra-operatively, the orthopedic oncology team completed the tumor resection while the plastic surgery team dissected out the brachial artery with elbow disarticulation, isolating the vascular pedicle. After amputation and vessel ligation, the wound measured 1200 cm^2^ with exposed subclavian vessels. On the back table, a cuff incision was made on the forearm at the distal wrist and extended vertically onto the dorsum. The flap was filleted open with subperiosteal elevation along the radius and ulna, which were then resected. Microvascular anastomosis proceeded in the standard fashion utilizing an end-to-side technique from the brachial artery into the subclavian artery and brachial vein into the subclavian vein. Perfusion was confirmed via indocyanine green fluorescence. The patient healed with no complications at with no cancer recurrence in the flap, but eventually succumbed to his disease.

## Case II

**Figure 2.**
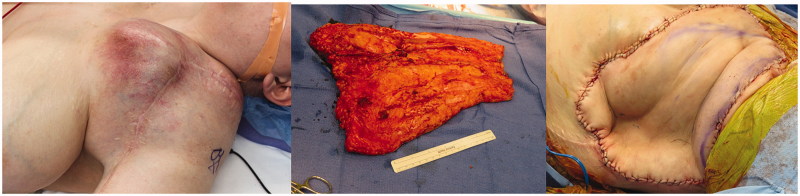
Case II pre-operative mass, intra-operative fasciocutaneous flap and post-operative result.

A 30-year-old female presented with a second recurrence of high-grade sarcoma of the left supraclavicular region (Figure 2). She had previously undergone radical resection of a proximal humeral osteosarcoma with implantation of a reverse total shoulder arthroplasty, complicated by recurrence two years later. This was radically resected with removal of the endoprosthesis and reconstructed with a pedicled latissimus dorsi muscle flap. A multi-disciplinary surgical team planned for re-resection and reconstruction with a free forearm fillet flap. Prior to tumor resection, the flap was elevated as a fasciocutaneous flap from distal to proximal utilizing a stocking-seam incision with inclusion of ulnar and radial arteries. As there was an expected delay with several hours of cold ischemia time, muscle was not included in the forearm fillet flap. Avoiding the inclusion of muscle reduced blood loss and allowed for an increased ischemia time with decreased reperfusion injury. The internal mammary artery and vein were chosen as recipient vessels as the tumor abutted the proximal axillary vessels. Radical tumor resection was performed resulting in forequarter amputation with a defect measuring 1000 cm^2^. After negative margins were confirmed, microvascular anastomosis was performed in an end-to-end manner, connecting the brachial artery and cephalic vein to the internal mammary artery and vein, respectively. The flap was trimmed and inset, and perfusion confirmed with indocyanine green fluorescent imaging. The patient healed with no complications at with no cancer recurrence in the flap, but eventually succumbed to her disease.

## Discussion

The concept of spare parts reconstruction is frequently utilized in traumatic extremity injuries, as it provides stable, vascularized coverage to a large wound while eliminating concomitant donor site morbidity [7,11–15]. However, the literature is limited in describing the safety of this technique in oncologic cases, specifically in cases of recurrent tumors. In these cases, local/regional and free tissue transfer procedures may have already been utilized, leaving a lack of donor sites. Patients with recurrent tumor also tend to have poor long-term survival rates, making the lack of functional donor-site morbidity an ideal option when considering fillet flaps.

In the upper extremity, the forearm can be easily circumferentially dissected, or ‘filleted’, to create a composite bulky flap [6,7,12]. This is especially advantageous in large defects with significant dead space. The vessels tend to be of large caliber with dependable perfusion to the flap; these sizeable vessels make for a technically easier anastomosis resulting in reduced operating time [2,5,14]. While the large flap size is advantageous, venous drainage can sometimes be limited. This can be resolved by ‘supercharging’ the venous outflow, in which case particular attention should be paid to vein selection. The venous drainage of the forearm is divided into superficial and deep systems. The cephalic and basilic veins are the major superficial veins of the forearm and the vena comitantes make up the deep supply. Ichinose et al. describes the efficacy of a dual venous anastomosis when separate venous systems are used, reporting a significant reduction in venous thrombosis and ultimately the risk of flap failure [16].

There are few absolute contraindications to the use of a free forearm fillet flap, including tumor extension into the distal forearm and prolonged ischemia time [5,12,17,18]. Patients undergoing a fillet flap for a severe traumatic injury are more susceptible to prolonged donor limb ischemia due to the inherent time spent during tumor resection. Ver Halen et al. describe attempts to limit ischemia by temporarily ‘banking’ the flap by vascular anastomosis to an alternative site, such as the groin, or re-vascularizing the tumor in the middle of the procedure before tumor resection was complete [19]. The senior authors modified the traditional fillet flap in the second case by taking it as a fasciocutaneous flap to allow for an increased ischemia time with decreased reperfusion injury. Relative contraindications include donor site infection, lymphedema and peripheral vascular disease [14,19]. These patients are at increased risk for infection, poor wound healing and edema [20].

Baccarani et al. described a treatment algorithm for the management of the amputated upper extremity, in which fillet flaps remain the first choice of reconstruction [21]. Alternatives to the fillet flap for forequarter amputations include rotational pedicled myocutaneous flaps from the distal humerus [22] and traditional free flaps from other areas of the body. Local options could include chimeric flaps based off the sub-scapular system to increase the surface area that can be covered [23]. However, for patients with oncologic history, local rotational flaps often are not available due to tumor burden, and traditional free flaps are avoided due to significant donor site morbidity. Fillet flaps not only have the benefit of no donor site morbidity but also can be performed with two teams with an acceptable complication profile [24].

In our series, both patients were reconstructed after resection of recurrent tumors. While these patients often have poor prognosis, both cases prove that use of the fillet flap is oncologically sound, as there was no cancer recurrence in either flap.

## Conclusion

The free forearm fillet flap is a viable option for reconstruction after forequarter amputation for recurrent upper extremity sarcomas. As few studies have reported its use in the management of large complex defects following high-grade tumor resection, this case series serves to expand the literature on an infrequent reconstructive technique.
